# Glass-Ceramic Materials with Luminescent Properties in the System ZnO-B_2_O_3_-Nb_2_O_5_-Eu_2_O_3_

**DOI:** 10.3390/molecules29153452

**Published:** 2024-07-23

**Authors:** Lyubomir Aleksandrov, Aneliya Yordanova, Margarita Milanova, Reni Iordanova, Peter Tzvetkov, Pavel Markov, Petia Petrova

**Affiliations:** 1 Institute of General and Inorganic Chemistry, Bulgarian Academy of Sciences, G. Bonchev, Str., bld. 11, 1113 Sofia, Bulgaria; lubomirivov@gmail.com (L.A.); margi@svr.igic.bas.bg (M.M.); reni@svr.igic.bas.bg (R.I.); tzvetkov@svr.igic.bas.bg (P.T.); pvlmarkov@svr.igic.bas.bg (P.M.); 2 Institute of Optical Materials and Technologies “Acad. Jordan Malinowski”, Bulgarian Academy of Sciences, blvd. Akad. G. Bonchev 109, 1113 Sofia, Bulgaria; petia@iomt.bas.bg

**Keywords:** glass ceramics, XRD, TEM, luminescence

## Abstract

In this paper, the crystallization behavior of 50ZnO:47B_2_O_3_:3Nb_2_O_3_:0.5Eu_2_O_3_ (G-0 h) glass has been investigated in detail by DSC, XRD and TEM analysis. The luminescent properties of the resulting glass-ceramics were also investigated. By XRD and TEM analysis, crystallization of several crystalline phases has been proved (α-Zn_3_B_2_O_6_, β-Zn_3_B_2_O_6_ and ZnNb_2_O_6_). By calculating crystal parameters, it was found that europium ions are successfully incorporated in the β-Zn_3_B_2_O_6_. Photo-luminescent spectra showed increased emission in the resulting glass-ceramic samples compared to the parent glass sample due to higher asymmetry of Eu^3+^ ions in the obtained crystalline phases. It was established that the optimum emission intensity is registered for glass-ceramic samples obtained after 25 h heat treatment of the parent glass.

## 1. Introduction

In recent years, a great deal of attention has been paid to rare earth-doped glass-ceramic materials, which play a crucial role in many optical applications such as up-conversion fibers, solid-state lasers, medical sensors, optical electronic chips, luminescence labels, optical amplifiers, 3D displays, etc. [1,2]. Most optical applications require transparency due to the reduced scattering effect because of the presence of nanocrystals inside glass-ceramics. In addition, nanocrystalline glass-ceramics might exhibit distinct optical properties in relation to pristine glasses when the rare earth ions occupy sites in the crystalline phase. In this case, an increase in emission intensities can occur due to the presence of rare earth ions in environments with lower phonon energy [3,4]. Transparent glass-ceramics possess excellent characteristics from both glasses and crystals and have no disadvantages of these two materials. Similar to glasses, glass-ceramics have a large capacity for accommodating an active rare earth dopant, are isotropic and have evenly distributed activators within their bodies. Similar to single crystals, glass-ceramics contain rare earth ions within strictly ordered ligand surroundings. As a result, the presence of a crystalline environment around a rare earth ion allows high absorption and emission cross-section reduction in the non-radiative relaxation process because of the lower phonon cut-off energy and tailoring of the ion–ion interaction by control of the rare earth ion partition [1,4]. There are data in the literature for photoluminescent glass-ceramics in many oxide and oxyfluoride systems such as: Tb^3+^, Eu^3+^-doped ZnO-B_2_O_3_; Tb^3+^, Eu^3+^, Er^3+^-doped ZnO-Al_2_O_3_-SiO_2_, ZnO-BaO-Na_2_O-SiO_2_, BaO-TiO_2_-SiO_2_ and CaF_2_-Al_2_O_3_-P_2_O_5_-SiO_2_; Re_2_O_3_-MoO_3_-B_2_O_3_ (Re = Sm, Gd, Tb, Dy and Eu); SiO_2_-BaF_2_-K_2_CO_3_-La_2_O_3_-Sb_2_O_3_-Eu_2_O_3_; Er^3+^-doped SrF_2_-ZnO-B_2_O_3_ [2,5,6,7,8,9,10].

The glass-ceramic materials are usually obtained by subsequent thermal treatment of a glass, first melted and annealed as usual. This conventional method relies on thermally induced phase separation and in situ crystallization processes, which are however very complex to experimentally control [4]. The choice of an appropriate glass composition is very important for luminescent glass-ceramics elaboration. The search for more efficient glass compositions and guiding structures for rare earth-doped glass-ceramics continues. 

More recently we have reported the preparation, structure and luminescence properties of niobium-modified zinc-borate glasses doped with Eu_2_O_3_ with compositions in mol% of 50ZnO:(50 − x)B_2_O_3_:xNb_2_O_5_:0.5Eu_2_O_3_, (x = 0, 1, 3 and 5 mol%) [11]. Through differential thermal analysis and density measurements, various physical properties such as molar volume, oxygen packing density and glass transition temperature were determined. IR and Raman spectra revealed that niobium ions enter the base zinc borate glass structure as NbO_4_ tetrahedra and NbO_6_ octahedra. It was found that the incorporation of Nb_2_O_5_ into Eu^3+^: ZnO:B_2_O_3_ glass creates more disordered and reticulated glass networks, which are favorable for doping with Eu^3+^ active ions. The luminescent properties of the obtained Eu^3+^-doped glasses revealed that they could be excited by 392 nm and exhibit pure red emission centered at 612 nm (^5^D_0_ → ^7^F_2_ transition). The introduction of niobium oxide into the ZnO:B_2_O_3_ glass enhances the luminescent intensity. The optimal concentration of Nb_2_O_5_ found to produce the most intensive red luminescence was 3 mol%.

In this work, we report the preparation and luminescent properties of glass-ceramic materials, obtained by controlled crystallization of glass with the composition in mol% of 50ZnO:47B_2_O_3_:3Nb_2_O_5_:0.5Eu_2_O_3_. The crystallization behavior of glass-ceramics was characterized by X-ray diffraction and transmission electron microscopy. Emission spectra were measured, and color coordinates of the materials were determined.

## 2. Results

### 2.1. Thermal Analysis and XRD Data

As mentioned at the Introduction section, in our previous study glasses with nominal composition 50ZnO:(50 − x)B_2_O_3_:xNb_2_O_5_:0.5Eu_2_O_3_, (x = 0, 1, 3 and 5 mol.%) were obtained and their luminescent properties were examined [11]. The optimal concentration of Nb_2_O_5_ to obtain the most intense red luminescence was found to be 3 mol% (x = 3). This was the reason for choosing the 50ZnO:47B_2_O_3_:3Nb_2_O_5_:0.5Eu_2_O_3_ (G-0 h) glass and investigating its crystallization ability in order to obtain glass-ceramics with enhanced luminescent properties compared to the initial glass. The glass-ceramics obtained after heat treatment of the parent glass for different times (5 h, 10 h, 15 h, 20 h, 25 h, 25 h, 30 h) are designated as GC-5 h, GC-10 h, GC-15 h, GC-20 h, GC-25 h, GC-30 h, respectively. 

**Figure 1:**
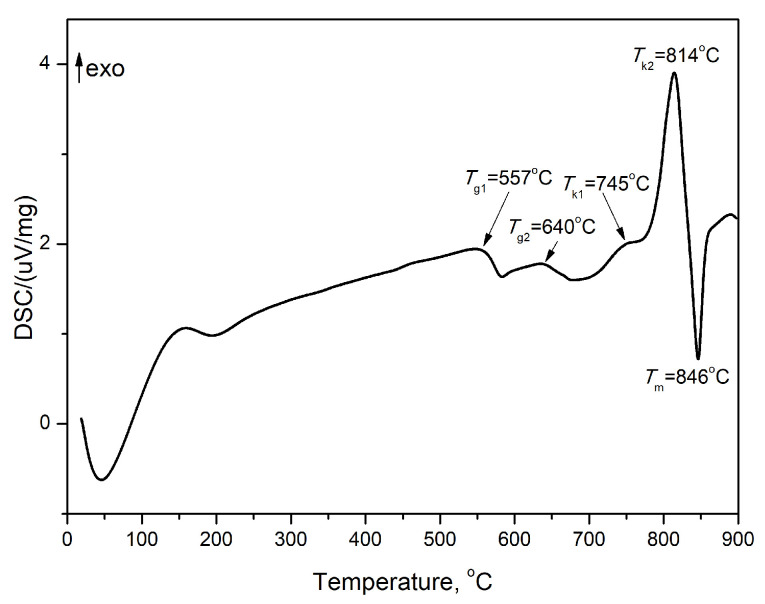
DSC curve of glass 50ZnO:47B_2_O_3_:3Nb_2_O5:0.5Eu_2_O_3_ (in mol %).

The DSC curve of the glass G-0 h is shown in Figure 1. It is seen that the DSC curve consists of two endothermic effects corresponding to the two glass transition temperatures (*T*_g1_ and *T*_g2_). This means that the synthesized glass consists of two amorphous phases due to the liquid phase separation. It is known that a large region of immiscibility exists in the binary system ZnO-B_2_O_3_ [6]. The maximum at the first endothermic effect (*T*_g1_) is called hysteresis peak, formed due to enthalpy relaxation. The next two exothermic peaks are due to the crystallization of the glass (*T*_kn_, where *n* = 1 and 2). The last endothermic effect corresponds to the melting temperature (*T*_m_). The obtained glass is characterized by a high glass transition temperature. The first *T*_g1_ value is 557 °C while the second *T*_g2_ value is 640 °C. The crystallization temperatures are as follows: 745 and 814 °C. The melting temperature is 846 °C. On the other hand, the thermal stability of the glass, i.e., ΔT = *T*_k1_ − *T*_g2_, is 105 °C.

No three component crystalline phases have been reported in the ternary ZnO-B_2_O_3_-Nb_2_O_5_ system. This means that the crystalline phases from the binary systems during the crystallization of glass G-0 h would be expected. In the phase-equilibrium diagram of the Nb_2_O_5_-B_2_O_3_ system, only one binary compound with composition 3Nb_2_O_5_:B_2_O_3_ exists, which melts incongruently [12]. There is also a large region of liquid phase separation in the range from 10 to 66 mol.% Nb_2_O_5_ [12]. There are two crystalline phases Zn_3_Nb_2_O_8_ and ZnNb_2_O_6_ (congruently melting) and a Nb-rich compound labeled Zn_2_Nb_34_O_87_ that melts incongruently in the binary system ZnO-Nb_2_O_5_ [13,14]. The third binary system is ZnO-B_2_O_3_, which is the richest in terms of crystalline phases. In the different references, the following crystalline phases have been announced: Zn_5_B_4_O_11_, ZnB_2_O_4_, Zn_3_B_2_O_6_, ZnB_4_O_7_ and Zn_4_B_6_O_13_ [14,15]. A large immiscibility region located above 50 mol% B_2_O_3_ is also reported [14,16]. 

**Figure 2:**
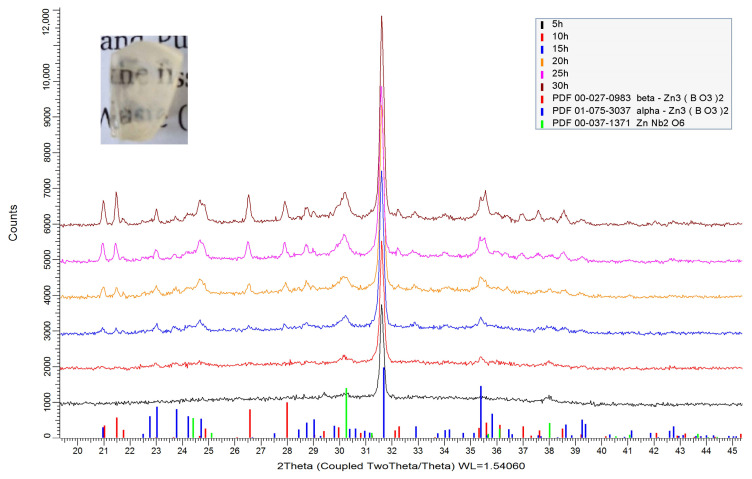
XRD patterns of the initial glass and of the glass-ceramic samples obtained.

The amorphous X-ray pattern of the glass 50ZnO:47B_2_O_3_:3Nb_2_O_5_:0.5Eu_2_O_3_ (G-0 h) was presented in our previous paper [11]. The parent glass G-0 h was heat treated for different periods of time (5, 10, 15, 20, 25 and 30 h) at 610 °C in order to avoid its full crystallization. The XRD patterns at room temperature of the glass-ceramics obtained are shown in Figure 2. The photograph of the translucent glass ceramic accomplished after 25 h heating of the initial glass at 610 °C is also shown as an inset of Figure 2. 

**Figure 3:**
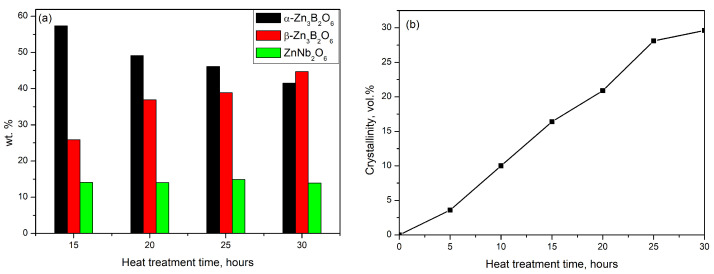
Comparisons of: (**a**) the ratio between obtained crystal phases in wt.%; (**b**) degree of crystallinity of glass-ceramic materials.

The weight percentage (wt.%) ratios of the crystalline phases formed are summarized in Figure 3a. For the GC-5 h and GC-10 h samples, it was impossible to determine the ratio between crystalline phases due to the low intensity of the diffraction lines. For sample GC-5 h, the predominated crystalline phase formed corresponds to α-Zn_3_B_2_O_6_ (ICDD PDF # 01-075-3037). A small amount of ZnNb_2_O_6_ (ICDD PDF # 00-037-1371) crystal phase was also detected. With increasing heat treatment time (15 h), the appearance of β-Zn_3_B_2_O_6_ (ICDD PDF #00-027-0983) was observed at the expense of both-α-Zn_3_B_2_O_6_ and the amorphous phase.

**Figure 4:**
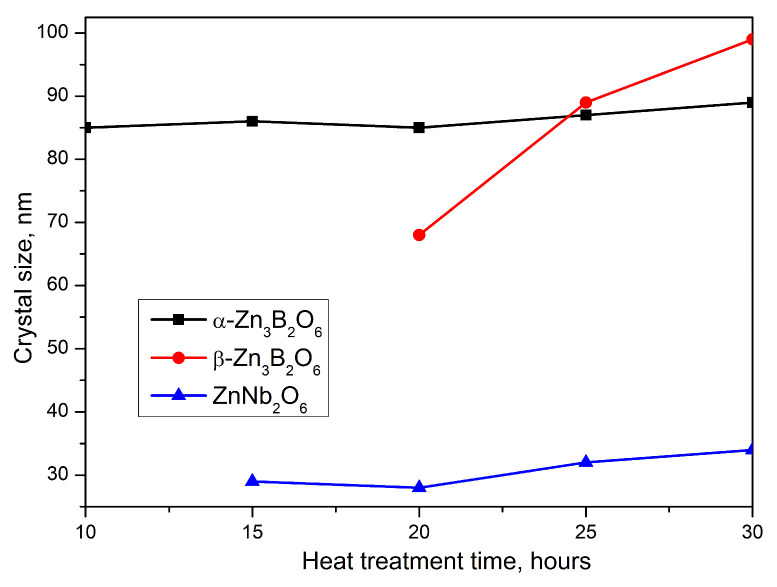
The crystallites size of the crystalline phases obtained in the crystallized samples.

X-ray analysis shows that β-Zn_3_B_2_O_6_ grows and dominates compared to the other crystalline phases in the glass-ceramic obtained after 30 h heat treatment of the parent glass (GC-30 h). Also, the amount of ZnNb_2_O_6_ crystalline phase does not change and remains within 14% in all glass-ceramic samples. Figure 3b traces the changes in the degree of crystallinity for all samples. It changes from 3.6% for GC-5 h and increases approximately up to 30% for GC-30 h. The crystallite sizes for all phases were calculated and the results are presented on Figure 4.

The particle sizes of β-Zn_3_B_2_O_6_ change from 68 to 99 nm. On the other hand, for α-Zn_3_B_2_O_6_ the crystal sizes do not change significantly (from 85 to 89 nm), and as well as for ZnNb_2_O_6_ (from 27 to 34 nm).

**Figure 5:**
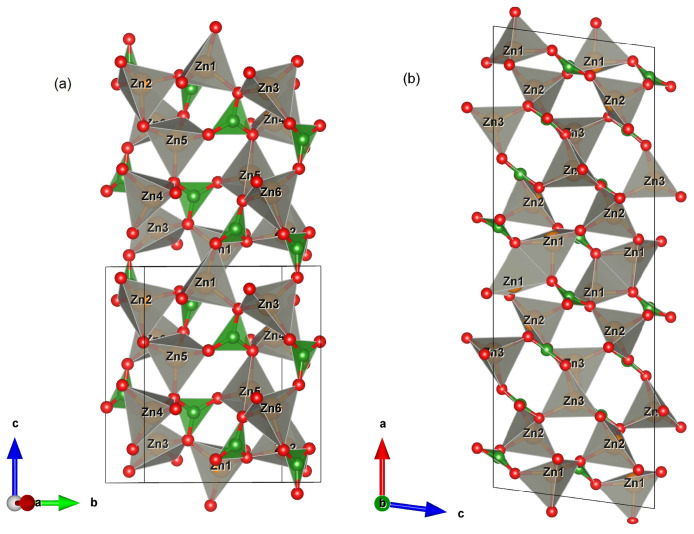
Polyhedral representation of the (**a**) α-Zn_3_B_2_O_6_ and (**b**) β-Zn_3_B_2_O_6_ crystal structures.

X-ray studies show that during the heat treatment of the glass the main crystalline phase is Zn_3_B_2_O_6_ in two polymorph modifications. For the first time Chen et al. [15] reported low-temperature α-Zn_3_B_2_O_6_ crystallized in a triclinic space group *P-1* with unit cell parameters a = 6.302(2) Å, b = 8.248(1) Å, c = 10.020(1) Å, α = 89.85(1)°, β = 89.79(1)°, γ = 73.25(1)°, V = 498.73 Å^3^ and Z = 4. The crystal structure of β-Zn_3_B_2_O_6_ was reported for the first time by Garcia-Blanco and Fayos [17] in monoclinic space group *Ic* (9) with unit cell parameters a = 23.40, b = 5.04, c = 8.38, β = 97.53°, V = 979.78 Å^3^ and Z = 8. Two years later, H. Baur and Tillmanns redetermined the crystal structure in the centrosymmetric space group *I2/c* (15) with the same unit cell parameters [18]. α-Zn_3_B_2_O_6_ represents a new structural type in which ZnO_4_ tetrahedra are connected to each other and also to BO_3_ flat triangles by common corners giving rise to a three-dimensional framework, while β-Zn_3_B_2_O_6_ is characterized by a three-dimensional network built from corner- and edge-sharing ZnO_4_ tetrahedra as well as corner sharing ZnO_4_ tetrahedra and BO_3_ triangles [15]. For comparison, the crystal structures of the two polymorphic modifications are presented in Figure 5. In the unit cell of the low-temperature modification, there are a total of six dis-tinct zinc atoms and four boron atoms, all located in Wyckoff position 2i. After the high-temperature transition, the symmetry increases to monoclinic, with all atoms now located in Wyckoff position 8f. In this polymorphic modification, the Zn1O_4_ tetrahedra form paired polyhedra connected via common edges (Figure 5b).

The second in quantity is the crystalline phase of ZnNb_2_O_6_. This compound crystallizes in orthorhombic space group *Pbcn* (60) with unit cell parameters: a = 14.208 Å, b = 5.726 Å, c = 5.04 Å, V = 410.03 Å^3^, Z = 4. The zinc niobate has a columbite-type structure in which Zn^2+^ and Nb^5+^ are in an octahedral environment. Each of the cations forms zigzag chains made up of octahedra connected by common edges and vertices along the [001] direction.

In this regard, the unit cell parameters and volume of the resulting crystalline phases during the thermal treatment of the glass were calculated and their values are presented in Table 1. 

As can be seen, a significant difference for the a-cell parameter of β-Zn_3_B_2_O_6_ was observed. For instance, the calculated a-parameter for β-Zn_3_B_2_O_6_ varies from 23.833 to 23.885 Å with increasing heat treatment time. Compared to the literature data, this parameter is 23.40 Å. Moreover, the volume of the unit cell increases from 979.78 Å^3^ for the referent β-Zn_3_B_2_O_6_ phase up to 985.1 Å^3^ for the resulting β-Zn_3_B_2_O_6_. For the other crystalline phases, no such changes are observed. This gives us reason to assume that the doped europium ions predominantly are accommodated in the β-Zn_3_B_2_O_6_ crystal structure.

**Figure 6:**
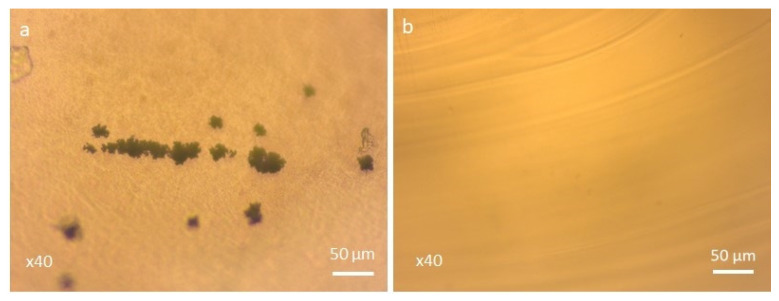
Optical microscopy images of glass-ceramic obtained by heat treatment of the glass for 25 h at 610 °C: (**a**) on the surface and (**b**) in the volume.

In order to clarify the mechanism of glass crystallization, we have performed optical microscopy measurements on the surface and in the volume of the resulting glass-ceramics and the results are shown on Figure 6. The images evidenced that surface crystallization takes place during the heat treatment of the glass. 

### 2.2. Luminescent Properties 

**Figure 7:**
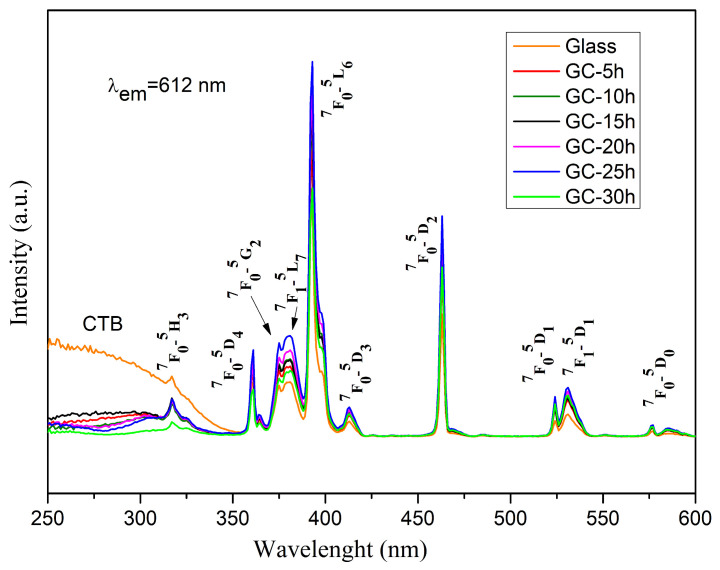
Excitation spectra of 50ZnO:47B_2_O_3_:3Nb_2_O_5_:0.5Eu_2_O_3_ glass and the corresponding glass-ceramics heat treated over different time durations.

In order to study the luminescent behavior of the synthesized glass and glass-ceramics, the emission (λ_ex_ = 392 nm) and excitation (λ_em_ = 612 nm) spectra of the obtained samples were measured. Figure 7 displays the room temperature PLE spectra of 0.5 mol% Eu^3+^ -doped 50ZnO:47B_2_O_3_:3Nb_2_O_5_ glass and glass-ceramics, recorded at 612 nm emission, corresponding to ^5^D_0_→^7^F_2_ transition of Eu^3+^ ions. 

The narrow peaks located in the spectral range 350–580 nm, are assigned to the characteristic 4f-4f transitions of Eu^3+^ ion, in particular from ^7^F_0_ → ^5^H_J_, ^7^F_0_ → ^5^D_4_, ^7^F_0_ → ^5^L_7_, ^7^F_0_ → ^5^L_6_, ^7^F_0_ → ^5^D_3_, ^7^F_0_ → ^5^D_2_, ^7^F_0_ → ^5^D_1_, ^7^F_1_ → ^5^D_1_, ^7^F_0_ → ^5^D_0_ at 317 nm, 361 nm, 380 nm, 392 nm, 413 nm, 463 nm, 523 nm, 531 nm and 576 nm, respectively [19]. The part of the spectra in the region of lower wavelengths, below 350 nm, is represented by a broad band due to the charge transfer transitions of the host absorbing groups, in particular Nb_2_O_n_ (O^2−^→Nb^5+^) [20] and ZnO_n_ (O^2−^→Zn^2+^) [21] and from oxygen 2p orbital to the empty 4f orbital of europium (O^2−^ → Eu^3+^) [22,23,24,25]. The appearance of this absorption in the excitation spectra when monitoring the Eu^3+^ emission at 612 nm is an indication of the occurrence of a charge transfer from the matrix, in this case from NbO_n_ and ZnO_n_ structural polyhedra to the rare earth Eu^3+^ ion, and previously has been shown to play an important role in the enhancement of the rare earth emission intensity [26,27,28,29,30]. This process is known as “host-sensitized” energy transfer. 

The intensity of the f-f peaks increases with the time of heat treatment up to 25 h and then decreases at 30 h. The same trend is observed in the emission spectra. This behavior is most likely caused by the change in active ion surroundings after glass crystallization and hence more efficient excitation of Eu^3+^ can be expected in the glass-ceramic samples [31]. The most intensive peak of the excitation spectra is located at 392 nm attributed to ^7^F_0_ → ^5^L_6_, followed by the ^7^F_0_ → ^5^D_2_ at 463 nm. These wavelengths can be used as an excitation source to register the emission spectra and are compatible with the commercially available near ultraviolet light-emitting diodes (LEDs) (250–400 nm) and blue LED chips (430–470 nm).

**Figure 8:**
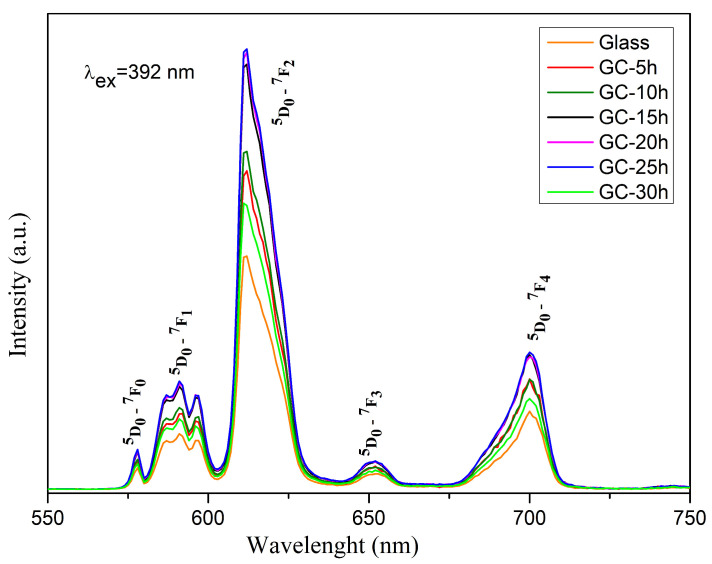
Emission spectra of 50ZnO:47B_2_O_3_:3Nb_2_O_5_:0.5Eu_2_O_3_ glass and the corresponding glass-ceramics heat treated over different time durations.

The room-temperature PL spectra are recorded in the range of 550–750 nm and are shown in Figure 8. The spectra are composed of the typical Eu^3+^ emission lines at 578 nm, 591 nm, 612 nm,651 and 700 nm due to the transitions from the ^5^D_0_ excited state to the ^7^F_0_, ^7^F_1_, ^7^F_2_, ^7^F_3_, ^7^F_4_ ground states of the Eu^3+^ ion, respectively [19]. 

The glass spectrum is characterized by the lowest luminescence intensity. After preparing glass-ceramic samples by heat treatment and crystallization at 610 °C, the emission intensity becomes stronger. The luminescent intensity of glass-ceramics increases with increasing heat treatment time up to 25 h. This behavior can be attributed to the incorporation of Eu^3+^ ions into the Zn_3_B_2_O_6_ crystal and, therefore, the enhancement of the spectral intensity is due to the change in the site symmetry around the Eu^3+^ ion. This fact is consistent with the X-ray structural data. A further increase in the treatment time to 30 h results in a sharp drop in emission intensity.

**Figure 9:**
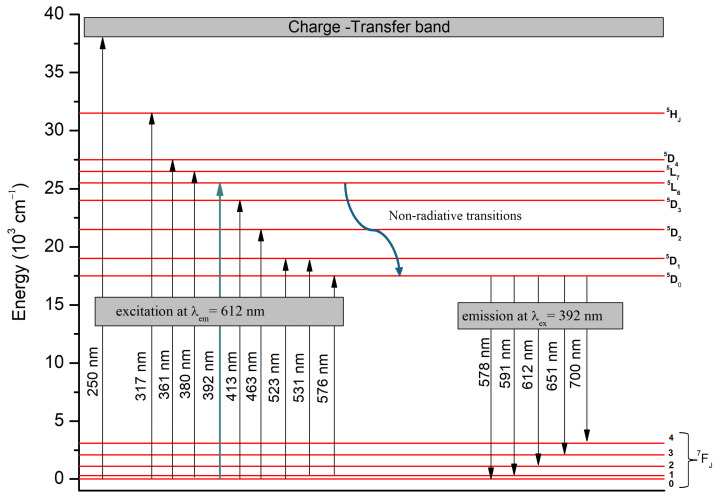
Energy-level diagram of Eu^3+^ ion in 50ZnO:47B_2_O_3_:3Nb_2_O_5_:0.5Eu_2_O_3_ glass and glass-ceramics.

The excitation and emission transitions of Eu^3+^ ions are illustrated in the schematic energy-level diagram in Figure 9.

As can be seen from Figure 8, the major emission line is located at 612 nm and is caused by the forced electric dipole transition (ED) ^5^D_0_ → ^7^F_2_. According to Judd–Ofelt theory, this transition is sensitive to the chemical bonds and site symmetry in the vicinity of Eu^3+^ ions, and it is most intensive when Eu^3+^ ions are occupying sites with non-inversion symmetry [32]. 

The magnetic dipole transition (MD) at 591 nm (^5^D_0_ → ^7^F_2_) is insensitive to the crystal field environment and can be used as a reference, since its intensity hardly varies with the change in the site symmetry around the Eu^3+^ ions [19,22]. An indication that Eu^3+^ ions are located in lattice sites without an inversion center is the more intensive ED emission compared to the MD one. Therefore, by calculating the intensity ratio of these two emissions (Table 2), known as the asymmetric ratio R, the degree of asymmetry in the local environment around the Eu^3+^ and the strength of Eu-O covalence in the different Eu^3+^-doped compounds can be studied. The lower the R value, the higher the local site symmetry around the active ion, and the lower Eu-O covalency and emission intensity [33]. Ratio values greater than 1 correspond to the location of Eu^3+^ ions in sites with lower symmetry, while values below 1 indicate that the active ion occupies high-symmetry sites. The calculated intensity ratios R (5.16–5.49) of the obtained samples are much higher than 1 (Table 2) indicating that they are characterized with high asymmetry in the vicinity of Eu^3+^ ions and strong Eu-O covalence. The asymmetry values of the glass-ceramics (5.21–5.49) are higher than the one of Eu^3+^-doped glass (5.16) indicating an enhanced emission intensity. Moreover, with increasing crystallinity, the asymmetry ratio increases gradually, up to the glass-ceramic sample with 25 h of thermal treatment (GC-25 h). This fact is directly related to the higher emission intensity of the heat-treated samples, as the higher the asymmetry the stronger the luminescent intensity. At 30 h heat treatment (CG-30 h), a decrease in R value is observed, which is consistent with the decrease in the emission intensity of this specimen.

Additionally, an indication of the low symmetry in the local environment around the Eu^3+^ is the appearance of the strictly forbidden ^5^D_0_ → ^7^F_0_ transition, which, as stated by Binnemans, shows that Eu^3+^ ions are located in sites with C_2v_, C_n_ or C_s_ symmetry [19]. Moreover, the splitting into three peaks of the ^5^D_0_ → ^7^F_1_ transition is also evidence that the symmetry of the Eu^3+^ sites in the studied glass and glass-ceramics are C_2v_ or lower [34].

**Figure 10:**
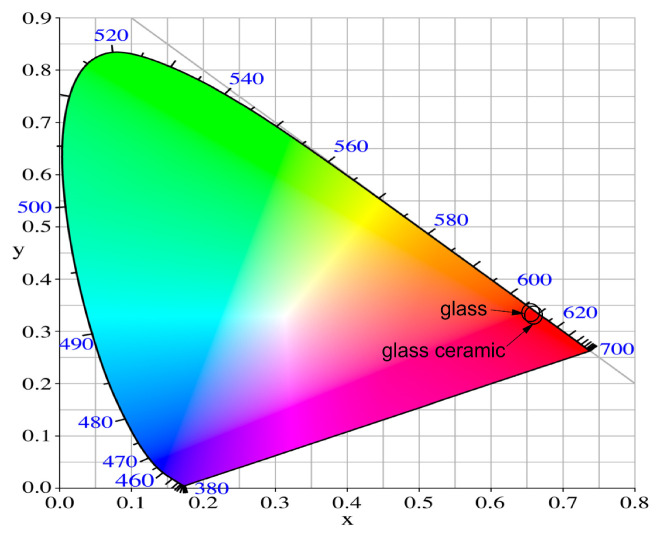
CIE chromaticity diagram of 50ZnO:47B_2_O_3_:3Nb_2_O_3_:0.5Eu_2_O_3_ glass and the corresponding glass-ceramic.

In order to estimate the actual emitted color, the Commission International de l’Eclairage (CIE) 1931 chromaticity diagram was used [35]. The chromaticity coordinates were calculated from the emission spectra by using the color calculator software SpectraChroma–Version 1.0.1 (a CIE coordinate calculator) [36]. The calculated values are listed in Table 3. The chromaticity coordinates of the glass and glass-ceramics lie in the red region in the CIE diagram (Figure 10). Their values are almost identical and accordingly are represented in the figure as one point. The closest to the CIE coordinate of standard red light (0.670, 0.330) and to the color coordinates of the commercial red phosphor Y_2_O_2_S:Eu^3+^ (0.658; 0.340) [37] is the glass sample (0.656, 0.343).

The obtained results indicate that the studied glass and glass-ceramics emit red light, which is very useful for the development of red-emitting solid-state devices. 

### 2.3. TEM Investigations and Density Measurment

**Figure 11:**
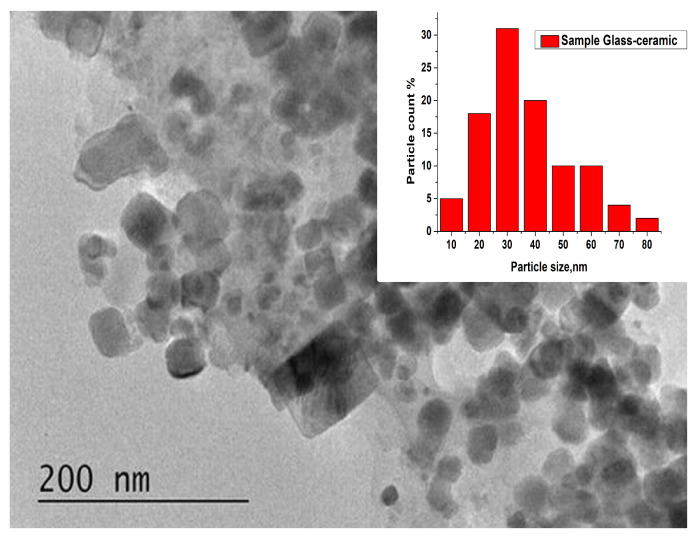
Bright field micrograph and particle size distribution (inset).

The morphology, particle size distribution and phase composition of the glass-ceramics sample obtained after 25 h heat treatment of the parent glass, characterized with the best luminescent properties, were investigated using TEM and HRTEM analytical methods. Morphology studies (Figure 11) show that the sample contains spherical and rectangular nanosized particles, as well as larger particles with sizes of about 80–90 nm. The inset in Figure 11 illustrates the particle size distribution.

**Figure 12:**
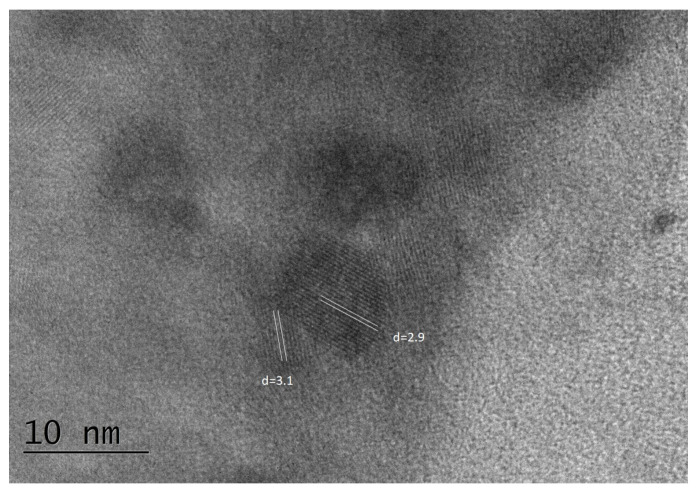
HRTEM picture of glass-ceramics obtained after 25 h heat treatment of the parent glass.

The average particle size of the glass-ceramics sample obtained after 25 h heat treatment of the parent glass is 33 nm. The particle sizes range between 10 and 80 nanometers, with some larger particles also present. The majority of the particles are in the range of 20–40 nm. HRTEM analysis (Figure 12) reveals the presence of both high-temperature monoclinic and low-temperature triclinic Zn_3_(BO_3_)_2_ phases in the sample, with cell parameters of a = 23.400, b = 5.040, c = 8.38 for the monoclinic phase, and a = 8.248, b = 10.020, c = 6.302 for the triclinic phase. The interplanar distances are d = 3.9 Å and d = 3.2 Å, respectively.

## 3. Discussion

In this paper, the glass crystallization behavior of 50ZnO:47B_2_O_3_:3Nb_2_O_3_:0.5Eu_2_O_3_ (G-0 h) glass has been investigated in detail by XRD and TEM analysis. XRD data show that the crystallization started after 5 h of heat treatment at 610 °C with separation of two crystalline phases-α-Zn_3_B_2_O_6_ and ZnNb_2_O_6_. After 15 h of heat treatment, β-Zn_3_B_2_O_6_ appears additionally and with further heating increases in amount at the expense of both α-Zn_3_B_2_O_6_ and glass phase. Comparative analysis of structural data of referent zinc borate and zinc niobate crystalline phases and glass-ceramics obtained here (see Table 1) shows more significant changes in both the parameter (a) and unit cell volume for β-Zn_3_B_2_O_6_ obtained from glass crystallization. This observation suggests that doped europium ions preferentially are accommodated in the β-Zn_3_B_2_O_6_. This suggestion is confirmed by the luminescence measurements as a drastic increase in the emission intensity of the GC-15 h was observed when β-Zn_3_B_2_O_6_ appears. With increasing time of heat treatment (20 and 25 h), the luminesce emission increases most probably because the amount of β-Zn_3_B_2_O_6_ also increases. However, 30 h of heat treatment leads to a decrease in luminescence intensity due to the emission quenching effect of europium ions. We assume that this quenching is a result of the decreased average distance between europium ions because of the increasing amount of β-Zn_3_B_2_O_6_. The enhanced photoluminescence emission in glass-ceramics samples compared to the glass is related to the covalence and structural changes in the vicinity of RE^3+^ ions (short range effect) [38]. The higher intensity of the ^5^D_0_ → ^7^F_2_ emission band in the glass-ceramics spectra as well as the higher values of the luminescence intensity ratio R compared to that of the parent glass can be related to the more asymmetrical coordination environment around Eu^3+^ ions in the glass-ceramics. On other hand, the increased emission intensity of Eu^3+^ ions in the crystallized samples compared to glass can be explained by the fact that in the glass-ceramics the crystalline particles are embedded in the amorphous matrix and more of them are separated from each other which improves the light scattering intensity from the free interfaces of the nanocrystallites [39].

## 4. Materials and Methods

Glass with nominal compositions 50ZnO:47B_2_O_3_:3Nb_2_O_5_:0.5Eu_2_O_3_ was obtained by applying the conventional melt-quenching method, using commercial powders of reagent grade Nb_2_O_5_ (Merck KGaA, Darmstadt, Germany), ZnO (Merck KGaA, Amsterdam, The Netherlands), H_3_BO_3_ (SIGMA-ALDRICH, St. Louis, MO, USA) and Eu_2_O_3_ (SIGMA-ALDRICH, St. Louis, MO, USA) as starting materials. The details of glass synthesis were given in ref. [5]. To prepare the glass-ceramics (GC), the precursor glass 50ZnO:47B_2_O_3_:3Nb_2_O_5_:0.5Eu_2_O_3_ was subjected to heat treatment at 610 °C for 5 h, 10 h, 15 h, 20 h, 25 h and 30 h. The glass transition (T_g_) temperature of the glass was determined by differential scanning calorimetry (DSC) using a Netzsch 404 Pegasus instrument, 2021 Selb, Germany, at a heating rate of 10 K/min in Ar flow of 10 mL/s, using corundum crucibles with lids. The XRD investigations were performed by using a Bruker D8 Advance X-ray powder diffractometer (Bruker-AXS, Karlsruhe, Germany) equipped with an X-ray tube with a copper anode (CuKα = 1.542 A, 40 kV and 40 mA) and a LynxEye position-sensitive detector (Bruker-AXS, Karlsruhe, Germany). The powder patterns were collected in the angular range 5.5–80.0° 2θ with a step of 0.03° 2θ and a total of 52.5 sec/step counting statistics, integrated over the whole area of the detector. The qualitative phase analysis was performed using the DIFFRAC.EVA v.4 software program [40] in combination with the ICDD PDF-2 (2021) reference database. The quantitative phase analysis, crystallite size, unit cell parameters and degree of crystallinity were determined using the Topas v.4.2 software program [41]. The crystal structure parameters for α-Zn_3_B_2_O_6_, β-Zn_3_B_2_O_6_ and ZnNb_2_O_6_ were taken from the literature cited in refs. [13,14,15,17,18]. Optical microscopy images were recorded using a Transmitted light microscope Primo Star equipped with 5-megapixel camera Axiocam 105 color with magnification 40× (ZEISS, Jena, Germany). The TEM observations were carried out using a transmission electron microscope JEM 2100 (JEOL, Tokio, Japan) with GATAN Orius 832 SC1000 CCD camera (AMETEK, Berwin, Pennsylvania, USA) at an accelerating voltage of 200 kV. The specimen for TEM investigation was prepared by grinding the sample in an agate mortar and then disintegrating it in the form of ethanol suspension by ultrasonic treatment for 6 min. A droplet of the suspension was coated on standard carbon film on a Cu grid. The size distribution of the particles was performed with the image-processing program ImageJ, and the measurements of the interplanar distances were performed with the specialized software Digital Micrograph (Version 2.31.734). Photoluminescence (PL) excitation and emission spectra at room temperature for all studied samples were measured with a FluoroLog3-22 spectrofluorometer, 2014 (Horiba Jobin-Yvon, Longjumeau, France).

## 5. Conclusions

In summary, Eu^3+^-doped 50ZnO:47B_2_O_3_:3Nb_2_O_5_:0.5Eu_2_O_3_ glass was prepared using melt-quenching method. Glass-ceramic materials with enhanced photoluminescence emission were obtained by controlled crystallization of the glass sample. Structural characterizations, i.e., TEM and XRD, verify the crystalline nature of the resulting glass-ceramics. X-ray studies show that during the heat treatment of the glass the main crystalline phase is Zn_3_B_2_O_6_ in two polymorph modifications. The second in quantity is the crystalline phase of ZnNb_2_O_6_. Morphology studies show that the glass-ceramic samples contain spherical and rectangular nanosized particles. The majority of the particles are in the range of 20–40 nm. Photoluminescence spectra revealed that the obtained glass-ceramics can emit red light under excitation of 392 nm originating from the dominant dipole transition ^5^D_0_ → ^7^F_2_ of Eu^3+^ ions. Based on the results from the emission spectra, it was established that the formation of β-Zn_3_B_2_O_6_:Eu^3+^ nanocrystals and the appropriate degree of crystallinity are decisive factors for improving the luminescence properties of the samples. The optical properties confirm that the obtained glass-ceramics are suitable hosts for the incorporation of Eu^3+^ ions and have potential applications in the field of red light-emitting diodes.

## Figures and Tables

**Table 1:** Calculated lattice parameter and unit cell volume for crystals resulting from heat treatment of glass. The unit cell angles are intentionally omitted. “n.a.” (not applicable).

Time	α-Zn_3_B_2_O_6_, a/b/c [Å], Volume [Å^3^]	β-Zn_3_B_2_O_6_ a/b/c [Å], Volume [Å^3^]	ZnNb_2_O_6_ a/b/c [Å], Volume [Å^3^]
30 h	6.311/8.267/10.035 500.9(2)	23.885/5.047/8.385 985.0(3)	14.219/5.706/5.066 411.0(4)
25 h	6.312/8.265/10.035 500.8(2)	23.885/5.048/8.383 985.1(4)	14.212/5.708/5.067 411.0(4)
20 h	6.310/8.264/10.031 500.5(3)	23.873/5.048/8.379 984.1(7)	14.216/5.701/5.067 410.7(6)
15 h	6.302/8.255/10.023 499.0(4)	23.833/5.039/8.365 978.7(7)	14.189/5.692/5.063 408.9(6)
10 h	n.a.	n.a.	n.a.
5 h	n.a.	n.a.	n.a.

**Table 2:** Relative luminescent intensity ratio (R) of the two transitions (^5^D_0_ → ^7^F_2_ )/(^5^D_0_ → ^7^F_1_ ) for 50ZnO:47B_2_O_3_:3Nb_2_O_3_:0.5Eu_2_O_3_ glass and glass-ceramics heat treated at different time durations.

Glass Composition	Relative Intensity Ratio, R
Glass 50ZnO:47B_2_O_3_:3Nb_2_O_3_:0.5Eu_2_O_3_	5.16
GC-5 h	5.28
GC-10 h	5.39
GC-15 h	5.42
GC-20 h	5.47
GC-25 h	5.49
GC-30 h	5.21

**Table 3:** CIE chromaticity coordinates of 50ZnO:47B_2_O_3_:3Nb_2_O_5_:0.5Eu_2_O_3_ glass and the corresponding glass-ceramics.

Glass Composition	Chromaticity Coordinates (x,y)
Glass 50ZnO:47B_2_O_3_:3Nb_2_O_3_:0.5Eu_2_O_3_	0.656, 0.343
GC-5 h	0.652, 0.347
GC-10 h	0.652, 0.348
GC-15 h	0.652, 0.348
GC-20 h	0.652, 0.348
GC-25 h	0.652, 0.348
GC-30 h	0.651, 0.348
NTSC standard for red light	0.670, 0.330
Y_2_O_2_S:Eu^3+^	0.658, 0.340

## Data Availability

Data are contained within the article.

## References

[1] Fedorov P.P., Luginina A.A.A., Popov I. (2015). Transparent oxyflouride glas ceramics. J. Fluor. Chem..

[2] Erth D. (2009). Photoluminescenec in Glass and Glass Ceramics. IOP Conf. Series Mater. Sci. Eng..

[3] Marcondes L.M., Evangelista R.O., Gonçalves R.R., de Camargo A.S.S., Manzani D., Nalin M., Cassanjes F.C., Poirier G.Y. (2019). Er^3+^-doped niobium alkali germanate glasses and glass-ceramics: NIR and visible luminescence properties. J. Non-Cryst. Solids.

[4] Ferrari M., Righini G.C. (2015). Glass-ceramic materials for guided-waste optics. Int. J. Appl. Sci..

[5] Ehrt D., Herrmann A., Tiegel M. (2011). Glasses and glass ceramics with blue, green and red photoluminescence. Phys. Chem. Glasses Eur. J. Glass Sci. Technol. B.

[6] Ehrt D. (2013). Zinc and manganese borate glasses—Phase separation, crystallization, photoluminescence and structure. Phys. Chem. Glasses Eur. J. Glass Sci. Technol. B.

[7] Wang Y., Honma T., Komatsu T. (2012). Synthesis and laser patterning of ferroelastic β’-Re_2_ (MoO_4_)_3_ crystals (Re: Sm. Gd, Tb, Dy) in rare earth molybdenum borate glasses. Mater. Chem. Phys..

[8] Wang Y., Honma T., Doi Y., Hinatsu Y., Komatsu T. (2013). Magnetism of β’-Gd_2_(MoO_4_)_3_ and photoluminescence of β’-Eu_2_(MoO_4_)_3_ crystallized in rare-earth molybdenum borate glasses. J. Ceram. Soc. Jpn..

[9] Biswas K., Sontakke A.D., Sen R., Annapurna K. (2012). Luminescence properties of dual valance Eu doped nano-crystalline BaF_2_ embedded glass-ceramics and observation of Eu^2+^→Eu^3+^ energy transfer. J. Fluoresc..

[10] Tanaka R., Kitagawa Y., Shinozaki K. (2023). Effect of adding Er^3+^ on the precipitated crystalline phase of SrF_2_-ZnO-B_2_O_3_ glass and upconversion luminescence. Opt. Mater..

[11] Iordanova R., Milanova M., Yordanova A., Aleksandrov L., Nedyalkov N., Kukeva R., Petrova P. (2024). Structure and Luminescent Properties of Niobium-Modified ZnO-B_2_O_3_:Eu^3+^ Glass. Mater..

[12] Levin E.M. (1966). Phase Equilibria in the System Niobium Pentoxide-Boric Acid. J. Res. Nationa Bur. Stand. A. Phys. Chem..

[13] Ballman A.A., Brown H. (1977). Czochralski growth in the zinc oxide-niobium pentoxide system. J. Cryst. Growth.

[14] Phase Equilibrium Diagrams, AcerS-NIST, CD-ROM Database, Version 3.1.0. https://ceramics.org/publications-resources/phase-equilibrium-diagrams/.

[15] Chen X., Hue H., Chang X., Zhang L., Zhao Y., Zuo J., Zang H., Xiao W. (2006). Syntheses and crystal structures of the α- and β-forms zinc orthoborate, Zn3B2O6. J. Alloys Compd..

[16] Dimitriev Y., Yordanova R., Aleksandrov L., Kostov K. (2009). Boromolybdate glasses containing rare earth oxides. Phys. Chem. Glasses Eur. J. Glass Sci. Technol. B.

[17] Garcia-Blanco S., Fayos J. (1968). The crystal structure of zinc orthoborate, Zn_3_(BO_3_)_2_. Z. Kristallogr..

[18] Baur W.H., Tillmanns E. (1970). The space group and crystal structure of trizinc diorthoborate. Z. Kristallogr..

[19] Binnemans K. (2015). Interpretation of europium (III) spectra. Coord. Chem. Rev..

[20] Zeng H., Song J., Chen D., Yuan S., Jiang X., Cheng Y., Chen G. (2008). Three-photon-excited upconversion luminescence of niobium ions doped silicate glass by a femtosecond laser irradiation. Opt. Express.

[21] Nimpoeno W.A., Lintang H.O., Yuliati L. (2020). Zinc oxide with visible light photocatalytic activity originated from oxygen vacancy defects. IOP Conf. Ser. Mater. Sci. Eng..

[22] Blasse G., Grabmaier B.C. (1994). Luminescent Materials.

[23] Hoefdraad H.E. (1975). The charge-transfer absorption band of Eu^3+^ in oxides. J. Solid State Chem..

[24] Parchur A.K., Ningthoujam R.S. (2012). Behaviour of electric and magnetic dipole transitions of Eu^3+^,^5^D_0_-^7^F_0_ and Eu-O charge transfer band in Li^+^ co-doped YPO_4_:Eu^3+^. RSC Adv..

[25] Mariselvam K., Liu J. (2021). Synthesis and luminescence properties of Eu^3+^ doped potassium titano telluroborate (KTTB) glasses for red laser applications. J. Lumin..

[26] Aleksandrov L., Milanova M., Yordanova A., Iordanova R., Nedyalkov N., Petrova P., Tagiara N.S., Palles D., Kamitsos E.I. (2023). Synthesis, structure and luminescence properties of Eu^3+^-doped 50ZnO.40B_2_O_3_.5WO_3_.5Nb_2_O_5_ glass. Phys. Chem. Glas. Eur. J. Glass Sci. Technol. B.

[27] Yordanova A., Aleksandrov L., Milanova M., Iordanova R., Petrova P., Nedyalkov N. (2024). Effect of the addition of WO_3_ on the structure and luminescent properties of ZnO-B_2_O_3_: Eu^3+^ glass. Molecules.

[28] Sreena T.S., Raj A.K., Rao P.P. (2022). Effects of charge transfer band position and intensity on the photoluminescence properties of Ca_1.9_M_2_O_7_: 0.1Eu^3+^ (M = Nb, Sb and Ta). Solid State Sci..

[29] Sun X.Y., Jiang D.G., Chen S.W., Zheng G.T., Huang S.M., Gu M., Zhao J.T. (2013). Eu^3+^- activated borogermanate scintillating glass with a high Gd_2_O_3_ content. J. Am. Ceram. Soc..

[30] Pang M., Liu X., Lin J. (2005). Luminescence properties of R_2_MoO_6_: Eu^3+^ (R = Gd, Y, La) phosphors prepared by Pechini sol-gel process. J. Mater. Res..

[31] Walas M., Lisowska M., Lewandowski T., Becerro A.I., Łapiński M., Synak A., Sadowski W., Kościelska B. (2019). From structure to luminescence investigation of oxyfluoride transparent glasses and glass-ceramics doped with Eu^3+^/Dy^3+^ ions. J. Alloys Compd..

[32] Shigeo S., William M. (1998). Phosphor Handbook.

[33] Dejneka M., Snitzer E., Riman R.E. (1995). Blue, green and red fluorescence and energy transfer of Eu^3+^ in fluoride glasses. J. Lumin..

[34] Binnemans K., Görller-Walrand C. (1996). Application of the Eu^3+^ ion for site symmetry determination. J. Rare Earths.

[35] Smith T., Guild J. (1931). The CIE colorimetric standards and their use. Trans. Opt. Soc..

[36] Paolini T.B. (2021). SpectraChroma (Version 1.0.1) [Computer Software]. https://zenodo.org/records/4906590.

[37] Trond S.S., Martin J.S., Stanavage J.P., Smith A.L. (1969). Properties of Some Selected Europium—Activated Red. J. Electrochem. Soc..

[38] Fu J., Kobayashi M., Sigimoto S., Parker J.M. (2009). Scintillation from Eu^2+^ in Nanocrystallized Glass. J. Am. Ceram. Soc..

[39] Aleksandrov L., Iordanova R., Dimitriev Y., Georgiev N., Komatsu T. (2014). Eu^3+^ doped 1La_2_O_3_:2WO_3_:1B_2_O_3_ glass and glass ceramic. Opt. Mater..

[40] (2014). DIFFRAC.EVA V.4.

[41] (2009). TOPAS V4.2.

